# Can We Predict Good Survival Outcomes by Classifying Initial and Re-Arrest Rhythm Change Patterns in Out-of-Hospital Cardiac Arrest Settings?

**DOI:** 10.7759/cureus.12019

**Published:** 2020-12-10

**Authors:** Heejun Shin, Giwoon Kim, Younghwan Lee, Hyungjun Moon, Hanjoo Choi, Choung Ah Lee, Hyuk Joong Choi, Yongjin Park, Kyoungmi Lee, Wonjung Jeong

**Affiliations:** 1 Emergency Medicine, Soonchunhyang University Hospital Bucheon, Bucheon, KOR; 2 Emergency Medicine, Soonchunhyang University Hospital Cheonan, Cheonan, KOR; 3 Emergency Medicine, Dankook University Hospital, Cheonan, KOR; 4 Emergency Medicine, Hallym University Dongtan Sacred Heart Hospital, Dongtan, KOR; 5 Emergency Medicine, Hanyang University Guri Hospital, Guri, KOR; 6 Emergency Medicine, Chosun University Hospital, Gwangju, KOR; 7 Emergency Medicine, Myongji Hospital, Goyang, KOR; 8 Emergency Medicine, Catholic University of Korea St. Vincent's Hospital, Suwon, KOR

**Keywords:** out-of-hospital cardiac arrest, advanced cardiac life support, cardiopulmonary resuscitation, re-arrest rhythm

## Abstract

Objective

The purpose of this study was to investigate whether a change in prehospital arrest rhythms could allow medical personnel to predict survival outcomes in patients who achieved a return of spontaneous circulation (ROSC) in the setting of out-of-hospital cardiac arrest (OHCA).

Methods

The design of this study was retrospective, multi-regional, observational, and cross-sectional with a determining period between August 2015 and July 2016. Cardiac arrest rhythms were defined as a shockable rhythm (S), which refers to ventricular fibrillation (VF) or pulseless ventricular tachycardia (pVT), and non-shockable rhythm (NS), which refers to pulseless electrical activity or asystole. Survival to admission, survival to discharge, and good cerebral performance category (CPC) (CPC 1 or 2) were defined as good survival outcomes.

Results

A total of 163 subjects were classified into four groups according to the rhythm change pattern: NS→NS (98), S→S (27), S→NS (23), and NS→S (15). NS→NS pattern was used as the reference in logistic regression analysis. In the case of survival to hospital admission, the odds ratio (OR) (95% CI) of the S→S pattern was the highest [12.63 (3.56-44.85), p: <0.001 by no correction] and [7.29 (1.96-27.10), p = 0.003 with adjusting]. In the case of survival to hospital discharge, the OR (95% CI) of the S→S pattern was the highest [37.14 (11.71-117.78), p: <0.001 by no correction] and [13.85 (3.69-51.97), p: <0.001 with adjusting]. In the case of good CPC (CPC 1 or 2) at discharge, the OR (95% CI) of the S→S pattern was the highest [96 (19.14-481.60), p: <0.001 by no correction] and [149.69 (19.51-1148.48), p: <0.001 with adjusting].

Conclusions

The S→S group showed the highest correlation with survival to hospital admission, survival to hospital discharge, and good CPC (CPC 1 or 2) at discharge compared to the NS→NS group. Verifying changes in initial cardiac arrest rhythm and prehospital re-arrest (RA) rhythm patterns after prehospital ROSC can help us predict good survival outcomes in the OHCA setting.

## Introduction

The increasing frequency of recurrent cardiac re-arrest (RA) in out-of-hospital cardiac arrest (OHCA) may cause medical staff to predict poor survival [1]. According to the literature, if the initial rhythm is shockable, then an episode of RA would also display a shockable rhythm [1]. On the other hand, if the initial rhythm is non-shockable it is likely that subsequent rhythm analysis following RA would stay as a non-shockable rhythm [1]. In 2015, the variable of "number of cardiac arrests attended" was newly added to the system description of the International Consensus on Cardiopulmonary Resuscitation consensus statement. This variable is one of the five OHCA Utstein definitions added to the Utstein Resuscitation Registry Templates for OHCA [2].

In South Korea, smart advanced life support (SALS), a state-led direct medical guidance-based emergency medical system (EMS) intervention, has been implemented beginning on August 1, 2015 [3]. SALS includes video medical guidance from a physician instructor to rescue personnel treating cardiac arrest patients and the subsequent implementation of specialized resuscitation in an OHCA situation [3].

The purpose of this study was to investigate whether changes in initial arrest rhythm and RA rhythm patterns are associated with survival to hospital admission, survival to hospital discharge, and good cerebral performance category (CPC 1 or 2) at discharge in OHCA patients who experienced prehospital RA following an episode of prehospital cardiac arrest with ROSC under SALS.

## Materials and methods

Study design and setting

The design of this study was retrospective, multi-regional, observational, and cross-sectional, which covered 12 South Korean cities in seven regions with a total of 4,369 km^2^ area and a population of about 12 million people. The SALS group performed this study from August 1, 2015, to the present [3]. The authors conducted a review of OHCA patients who received SALS from August 1, 2015, to July 31, 2016, by examining first aid and hospital medical records in the SALS registry [3].

SALS, SALS data source, collection, and registry

The National Emergency Medical Center (NEMC) and National Fire Agency (NFA) in South Korea have developed a specific visual direct medical oversight for OHCA called SALS [3,4]. SALS is executed by two teams consisting of paramedics and emergency medical technicians (EMTs) who are dispatched to the scene of a cardiac arrest call and arrive at the scene under a two-tiered response system [4]. Both teams move to the scene and do the work in a first-come-first-served way where the first-comer team performs the high-quality basic life support (BLS) and both teams, after incorporating the second-comer team, expand actions from BLS to field advanced cardiovascular life support (ACLS) under direct medical direction by a physician instructor [4]. The physician instructor determines the use of resuscitative drugs, such as epinephrine or amiodarone, and monitors the overall process of SALS to enhance the linkage between BLS and field ACLS [4]. After the resuscitation, paramedics and medical directors complete the cardiopulmonary resuscitation (CPR) chart of SALS patients, which is designed by the international Utstein style for cardiac arrest that is electronically stored in each provincial EMS headquarters and managed by the NFA [2,5]. CPR recording chart (or SALS chart), which is configured in intervals of one minute, can be used for recording up to 60 minutes of treatment. NEMC and physicians responsible for database perform quality control by completing the record on outcomes to manage the missing data [3,4]

Participants

The inclusion criteria included non-traumatic adult OHCA patients aged over 18 years with prehospital ROSC and at least one RA in the prehospital phase after prehospital ROSC under the SALS registry.

Study protocol

**Figure 1:**
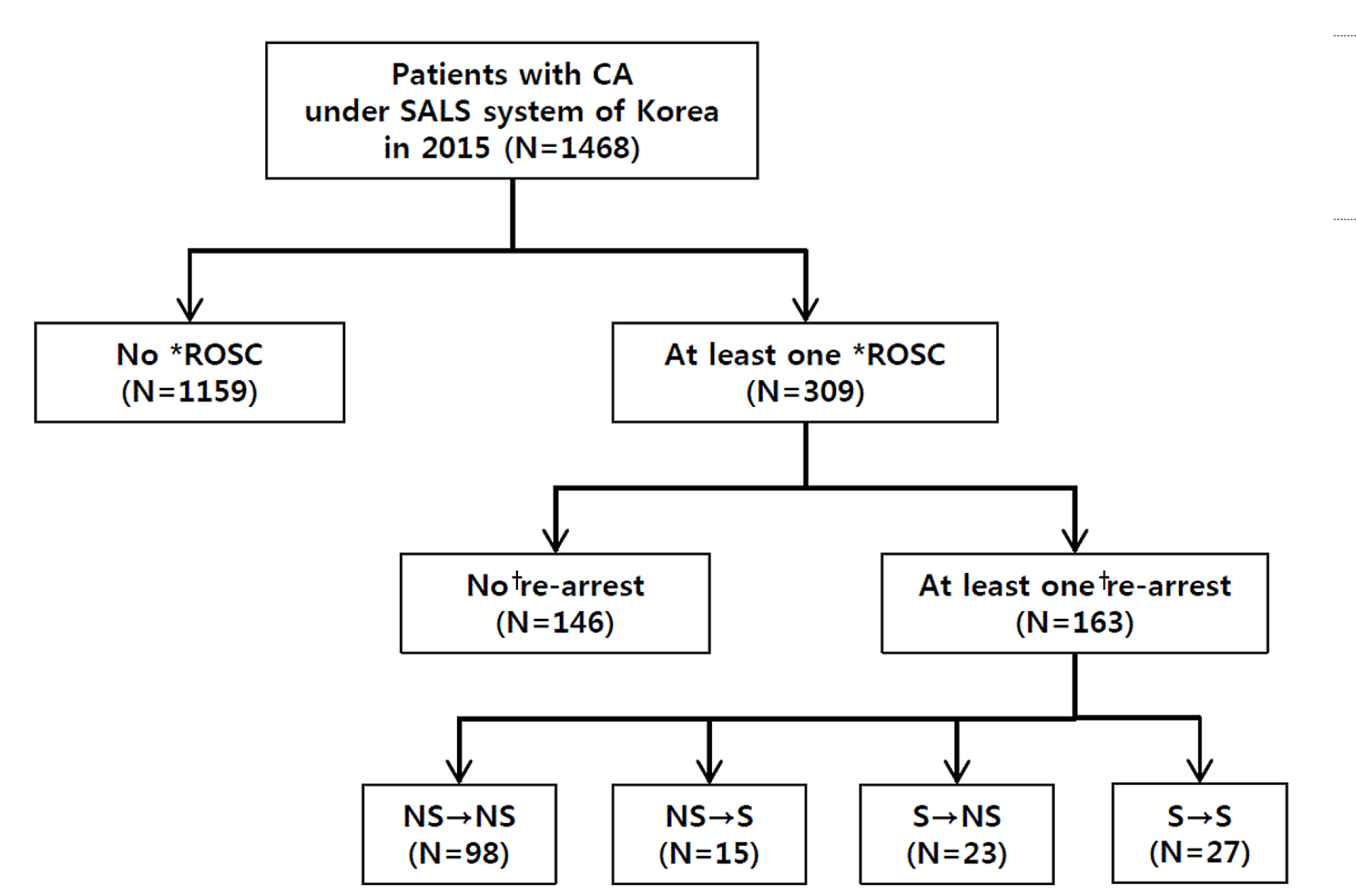
Flow chart detailing the selection of the study population *prehospital any ROSC; ^†^prehospital re-arrest CA: cardiac arrest; S: shockable; NS: non-shockable rhythm; ROSC: return of spontaneous circulation

The total number of non-traumatic OHCA patients who received SALS was 1,468. Of these, 163 adult patients over 18 years of age who experienced RA during the prehospital after prehospital ROSC were selected as the final study subjects (Figure 1).

Independent variables

Independent variables included age, sex, underlying disease [hypertension (HTN), diabetes mellitus (DM), lung disease, tuberculosis, hepatitis, heart disease, allergy, stroke, cancer, and other], occurrence site (public place, home, nursing facility, ambulance, and other), witnessed cardiac arrest, bystander CPR, bystander automated external defibrillator (AED) application, Utstein style, awareness by the management center, backup assistant ambulance, securing an advanced airway, and intravenous (IV) success. Utstein style was defined as the universal measurement tool for the CPR effect that meets criteria including cardiogenic arrest, witnessed cardiac arrest, and shockable rhythm [5].

Outcome variables

Outcome variables included survival to hospital admission, survival to hospital discharge, and good CPC (CPC 1 or 2) at discharge.

The three groups categorized according to prehospital ROSC and prehospital RA status

Three groups were categorized according to prehospital ROSC and prehospital RA status for comparing characteristics as follows: no prehospital any ROSC, prehospital RA after prehospital any ROSC, and no prehospital RA after prehospital any ROSC. RA rhythm was defined as the firstly identified cardiac arrest rhythm after ROSC in EMS.

Classification of four types of cardiac arrest rhythm change pattern in prehospital RA after prehospital any ROSC

- Four groups: NS→NS, NS→S, S→NS, and S→S

- Patterns of change between initial arrest rhythms and RA rhythms recognized by EMS personnel

- Shockable rhythm (S) was defined as pulseless ventricular tachycardia (pVT) or ventricular fibrillation (VF)

- Non-shockable rhythm (NS) was defined as asystole or pulseless electrical activity (PEA)

Good survival outcome

Survival to hospital admission, survival to hospital discharge, and good CPC (CPC 1 or 2) at discharge were set as good survival outcomes in this study. The definition of good CPC (CPC 1 or 2) at discharge in detail is as follows: 

- Good CPC was defined as CPC 1 or 2 [5]

- CPC was categorized into five types as follows [5]:

CPC 1: good cerebral performance

CPC 2: moderate cerebral disability

CPC 3: severe cerebral disability

CPC 4: coma or vegetative state

CPC 5: brain death

Study analysis

Characteristics According to Prehospital Any ROSC and Prehospital RA status

The authors examined the characteristics of independent variables that differed among the groups characterized by no prehospital ROSC, RA after prehospital ROSC, and no RA after prehospital ROSC.

Characteristics According to the Change in Cardiac Rhythm Pattern in Prehospital RA Patients After Prehospital Any ROSC

The characteristics of the independent variables among the four groups (NS→NS, NS→S, S→NS, and S→S) in prehospital RA patients after prehospital any ROSC were analyzed and compared.

Logistic Regression Analysis for Good Survival Outcomes in Prehospital RA Patients After Prehospital Any ROSC

- Logistic regression analysis with no correction: the odds ratio (OR) (95% CI) for good CPC (CPC 1 or 2) at discharge using only the arrest rhythm change pattern of NS→NS, NS→S, S→NS, and S→S was calculated.

- Logistic regression analysis with adjusting independent variables: the OR (95% CI) was derived by applying the adjusted variables for survival to hospital admission, survival to hospital discharge, and good CPC (CPC 1 or 2) at discharge, as well as the arrest rhythm change pattern. Univariate logistic regression analysis was performed for all independent variables for good CPC (CPC 1 or 2) at discharge, and variables with p-values of <0.05 were included among the adjustment parameters.

Statistical analysis

Data were analyzed using SPSS Statistics for Windows 21.0 (IBM, Armonk, NY). Data were reported as mean ± standard deviation for continuous variables and n (%) for categorical variables. P-values were calculated by one-way analysis of variance (ANOVA) or the Kruskal-Wallis test for continuous variables, and the chi-square test or Fisher's exact test for categorical variables. Logistic regression analysis including variable calibration was used. A p-value of <0.05 was defined as statistically significant. The backward selection was used for the logistic regression analysis in SPSS.

## Results

Characteristics according to prehospital any ROSC and prehospital RA status

The survival outcome of the "no prehospital any ROSC" group was the worst, and the "prehospital RA after prehospital any ROSC" group had a worse outcome than the "no prehospital RA after prehospital any ROSC" group (p: <0.05) (Table 1). There were significant differences among the three groups in terms of age, sex, the presence of DM and other diseases, occurrence site, witnessed cardiac arrest, Utstein style, awareness of the management center, securing an advanced airway, venous access, time to the first injection of epinephrine (TFIE), response time interval (RTI), on-scene time interval (STI), total transport time (TTT), and total prehospital time (TPT) (p: <0.05) (Table 1).

**Table 1:** Characteristics according to prehospital any ROSC and prehospital RA status *ROSC: prehospital any ROSC; ^†^RA: prehospital RA; ^‡^Utstein style: universal measurement tool for the CPR effect that meets criteria including cardiogenic arrest, witnessed cardiac arrest, and shockable rhythm ROSC; return of spontaneous circulation; RA: re-arrest; CPR: cardiopulmonary resuscitation; AED: automated external defibrillator; HTN: hypertension; DM: diabetes mellitus; CPC: cerebral performance category; IV: intravenous; SD: standard deviation Data are reported as mean ± standard deviation for continuous variables and number (%) for categorical variables. P-values were calculated by one-way ANOVA or the Kruskal-Wallis test for continuous variables, and the chi-square test or Fisher's exact test for categorical variables

Variable	Total (n=1,468)		No *ROSC (n=1,159)	^†^RA after *ROSC (n=163)	No ^†^RA after *ROSC (n=143)	P-value
							Age (years), mean ± SD	67.6 ± 15.4		69.3 ± 14.9	62.8 ± 15.4	59.9 ± 15.7	<0.001
Males, n (%)	1,010 (68.8%)		775 (66.9%)	123 (75.5%)	112 (76.7%)	0.008
Underlying disease						
HTN, n (%)	403 (27.5%)		332 (28.6%)	40 (24.5%)	31 (21.2%)	0.113
DM, n (%)	290 (19.8%)		245 (21.1%)	29 (17.8%)	16 (11.0%)	0.012
Lung disease, n (%)	76 (5.2%)		67 (5.8%)	4 (2.5%)	5 (3.4%)	0.12
Tuberculosis, n (%)	3 (0.2%)		3 (0.3%)	0 (0.0%)	0 (0.0%)	0.67
Hepatitis, n (%)	1 (0.1%)		1 (0.1%)	0 (0.0%)	0 (0.0%)	0.875
Heart disease, n (%)	240 (16.3%)		188 (16.2%)	25 (15.3%)	27 (18.5%)	0.731
Allergy, n (%)	1 (0.1%)		0 (0.0%)	1 (0.6%)	0 (0.0%)	0.211
Stroke, n (%)	98 (6.7%)		79 (6.8%)	11 (6.7%)	8 (5.5%)	0.83
Cancer, n (%)	122 (8.3%)		105 (9.1%)	10 (6.1%)	7 (4.8%)	0.12
Other, n (%)	525 (35.8%)		439 (37.9%)	52 (31.9%)	34 (23.3%)	0.001
Occurrence site						<0.001
Public place, n (%)	268 (18.3%)		178 (15.4%)	50 (30.7%)	40 (27.4%)	
Home, n (%)	1,049 (71.5%)		848 (73.2%)	105 (64.4%)	96 (65.8%)	
Nursing facility, n (%)	133 (9.1%)		122 (10.5%)	4 (2.5%)	7 (4.8%)	
Ambulance, n (%)	7 (0.5%)		2 (0.2%)	2 (1.2%)	3 (2.1%)	
Other, n (%)	11 (0.7%)		9 (0.8%)	2 (1.2%)	0 (0.0%)	
Witnessed cardiac arrest, n (%)	715 (48.7%)		517 (44.6%)	97 (59.5%)	101 (69.2%)	<0.001
Bystander CPR, n (%)	1,025 (69.8%)		803 (69.3%)	110 (67.5%)	112 (76.7%)	0.144
Bystander AED application, n (%)	37 (2.5%)		24 (2.1%)	6 (3.7%)	7 (4.8%)	0.085
^‡^Utstein style, n (%)	207 (14.1%)		97 (8.4%)	46 (28.2%)	64 (43.8%)	<0.001
Awareness by the management center, n (%)	1,129 (76.9%)		912 (78.7%)	110 (67.5%)	107 (73.3%)	0.004
Backup assistant ambulance, n (%)	1,433 (97.6%)		1,131 (97.6%)	162 (99.4%)	140 (95.9%)	0.131
Securing advanced airway, n (%)	1,402 (95.5%)		1,114 (96.1%)	160 (98.2%)	128 (87.7%)	<0.001
IV success, n (%)	1,087 (74.0%)		828 (71.4%)	152 (93.3%)	107 (73.3%)	<0.001
Outcome						
Survival to hospital admission, n (%)	274 (18.7%)		79 (6.8%)	81 (49.7%)	114 (78.1%)	<0.001
Survival to hospital discharge, n (%)	137 (9.3%)		15 (1.3%)	36 (22.1%)	86 (58.9%)	<0.001
Good CPC (CPC 1 or 2) at discharge, n (%)	97 (6.6%)		2 (0.2%)	27 (16.6%)	68 (46.6%)	<0.001

Characteristics according to cardiac rhythm pattern in prehospital RA patients after prehospital any ROSC

**Figure 2:**
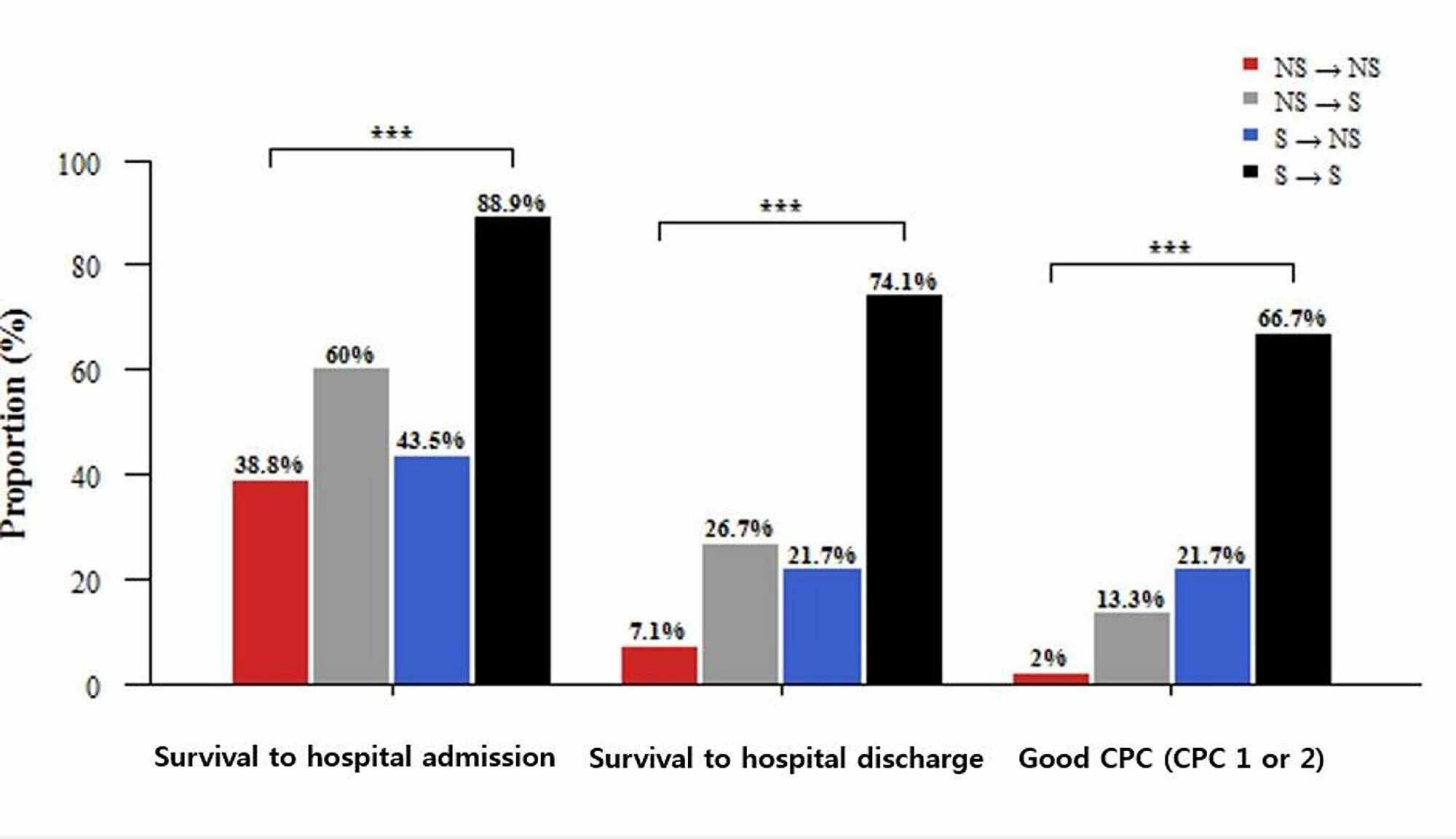
Survival outcomes according to rhythm pattern in prehospital RA patients after prehospital any ROSC ***P: <0.001 by Fisher's exact test NS: non-shockable; S: shockable; RA: re-arrest; ROSC: return of spontaneous circulation; CPC: cerebral performance category

The mean age among participants was was 62.8 ± 15.4 years. The S→NS and S→S groups were younger than the NS→NS and NS→S groups (p: <0.05) (Table 2). Among the 163 patients, the incidence of initial arrest rhythm was as follows (from high to low): PEA, 46.0%, 75/163; VF, 27.0%, 44/163; asystole, 23.3%, 38/163; and VT, 3.7%, 6/163 (p: <0.05) (Table 2). The order of RA rhythm incidence was asystole (40.5%, 66/163), PEA (33.7%, 55/163), VF (20.3%, 33/163), and VT (5.5%, 9/163) (p: <0.05) (Table 2). The ratio of asystole and VT among the 163 patients increased from initial arrest rhythm to RA rhythm, while the ratio of PEA and VF decreased (p: <0.05) (Table 2). Fisher's exact test revealed that the S→S group had the highest rates of survival to hospital admission, survival to hospital discharge, and good CPC (CPC 1 or 2) at discharge among all four groups (p: <0.05) (Table 2, Figure 2). Regarding survival to hospital admission and survival to hospital discharge, the rates of change in initial arrest rhythm and RA rhythm were (in descending order) S→S, NS→S, S→NS, and NS→NS (p: <0.05) (Table 2, Figure 2). In patients with good CPC (CPC 1 or 2) at discharge, the rates of change in initial arrest rhythm and RA rhythm were (from low to high) S→S, S→NS, NS→S, and NS→NS (p: <0.05) (Table 2, Figure 2).

**Table 2:** Characteristics according to cardiac rhythm pattern in prehospital RA patients after prehospital any ROSC †Utstein style: universal measurement tool for the CPR effect that meets criteria including cardiogenic arrest, witnessed cardiac arrest, and shockable rhythm NA: not available or not accountable; ROSC: return of spontaneous circulation; RA: re-arrest; PEA: pulseless electrical activity; VF: ventricular fibrillation; pVT: pulseless ventricular tachycardia; initial arrest rhythm: asystole, PEA, VF, and pVT; RA rhythm: asystole, PEA, VF, and pVT; CPR: cardiopulmonary resuscitation; AED: automated external defibrillator; HTN: hypertension; DM: diabetes mellitus; EMT: emergency medical technician; TFIE: time to the first injection of epinephrine [time interval from initial patient contact of the emergency medical system (EMS) to intravenous (IV) epinephrine injection]; RTI: response time interval (time interval from awareness of the victim by the bystander to call to the EMS); STI: scene time interval; TTT: total transport time; TPT: total prehospital time (the sum of RTI, STI, and TTT); TOBC: time to the onset of bystander CPR (time interval from awareness of the victim to the onset of bystander CPR); TOEC: time to the onset of CPR by the EMT (time interval from awareness of the victim to the onset of CPR by the EMT); TOC: time to the onset of CPR performed by the EMT who was activated by a 119 EMS call in South Korea; CPC: cerebral performance category; SD: standard deviation Data are reported as mean ± standard deviation for continuous variables and number (%) for categorical variables. P-values were calculated by one-way ANOVA or the Kruskal-Wallis test for continuous variables and the chi-square test or Fisher's exact test for categorical variables

Variable	Total (n=163)	NS→NS (n=98)	NS→S (n=15)	S→NS (n=23)	S→S (n=27)	P-value
							Age (years), mean ± SD	62.8 ± 15.4	66.0 ± 14.2	67.9 ± 10.9	53.0 ± 19.2	56.7 ± 12.9	0.001
Males, n (%)	123 (75.5%)	66 (67.3%)	11 (73.3%)	20 (87.0%)	26 (96.3%)	0.009
Underlying disease						
HTN, n (%)	40 (24.5%)	24 (24.5%)	4 (26.7%)	4 (17.4%)	8 (29.6%)	0.789
DM, n (%)	29 (17.8%)	23 (23.5%)	1 (6.7%)	4 (17.4%)	1 (3.7%)	0.069
Lung disease, n (%)	4 (2.5%)	3 (3.1%)	0 (0.0%)	1 (4.3%)	0 (0.0%)	0.67
Tuberculosis, n (%)	0 (0.0%)	0 (0.0%)	0 (0.0%)	0 (0.0%)	0 (0.0%)	NA
Hepatitis, n (%)	0 (0.0%)	0 (0.0%)	0 (0.0%)	0 (0.0%)	0 (0.0%)	NA
Heart disease, n (%)	25 (15.3%)	13 (13.3%)	5 (33.3%)	4 (17.4%)	3 (11.1%)	0.211
Allergy, n (%)	1 (0.6%)	1 (1.0%)	0 (0.0%)	0 (0.0%)	0 (0.0%)	0.881
Stroke, n (%)	11 (6.7%)	8 (8.2%)	1 (6.7%)	1 (4.3%)	1 (3.7%)	0.821
Cancer, n (%)	10 (6.1%)	8 (8.2%)	0 (0.0%)	1 (4.3%)	1 (3.7%)	0.555
Other, n (%)	52 (31.9%)	30 (30.6%)	5 (33.3%)	10 (43.5%)	7 (25.9%)	0.582
Occurrence site						0.194
Public place, n (%)	50 (30.7%)	21 (21.4%)	8 (53.3%)	7 (30.4%)	14 (51.9%)	
Home, n (%)	105 (64.4%)	70 (71.4%)	7 (46.7%)	15 (65.2%)	13 (48.1%)	
Nursing facility, n (%)	4 (2.5%)	3 (3.1%)	0 (0.0%)	1 (4.3%)	0 (0.0%)	
Ambulance, n (%)	2 (1.2%)	2 (2.0%)	0 (0.0%)	0 (0.0%)	0 (0.0%)	
Other, n (%)	2 (1.2%)	2 (2.0%)	0 (0.0%)	0 (0.0%)	0 (0.0%)	
Initial arrest rhythm						<0.001
Asystole, n (%)	38 (23.3%)	36 (36.7%)	2 (13.3%)	0 (0.0%)	0 (0.0%)	
PEA, n (%)	75 (46.0%)	62 (63.3%)	13 (86.7%)	0 (0.0%)	0 (0.0%)	
VF, n (%)	44 (27.0%)	0 (0.0%)	0 (0.0%)	19 (82.6%)	25 (92.6%)	
pVT, n (%)	6 (3.7%)	0 (0.0%)	0 (0.0%)	4 (17.4%)	2 (7.4%)	
RA rhythm						<0.001
Asystole, n (%)	66 (40.5%)	53 (54.1%)	0 (0.0%)	13 (56.5%)	0 (0.0%)	
PEA, n (%)	55 (33.7%)	45 (45.9%)	0 (0.0%)	10 (43.5%)	0 (0.0%)	
VF, n (%)	33 (20.3%)	0 (0.0%)	10 (66.7%)	0 (0.0%)	23 (85.1%)	
pVT, n (%)	9 (5.5%)	0 (0.0%)	5 (33.3%)	0 (0.0%)	4 (14.9%)	
Witnessed cardiac arrest, n (%)	97 (59.5%)	52 (53.1%)	12 (80.0%)	14 (60.9%)	19 (70.4%)	0.13
Bystander CPR, n (%)	110 (67.5%)	63 (64.3%)	9 (60.0%)	16 (69.6%)	22 (81.5%)	0.348
Bystander AED application, n (%)	6 (3.7%)	4 (4.1%)	1 (6.7%)	0 (0.0%)	1 (3.7%)	0.729
^†^Utstein style, n (%)	46 (28.2%)	7 (7.1%)	8 (53.3%)	13 (56.5%)	18 (66.7%)	<0.001
Awareness of the management center, n (%)	110 (67.5%)	66 (67.3%)	10 (66.7%)	18 (78.3%)	16 (59.3%)	0.561
Backup assistant ambulance, n (%)	162 (99.4%)	98 (100.0%)	15 (100.0%)	23 (100.0%)	26 (96.3%)	0.167
Securing an advanced airway, n (%)	160 (98.2%)	97 (99.0%)	15 (100.0%)	23 (100.0%)	25 (92.6%)	0.127
IV success, n (%)	152 (93.3%)	94 (95.9%)	15 (100.0%)	22 (95.7%)	21 (77.8%)	0.005
Time-related factors						
TFIE (min), mean ± SD	14.0 ± 7.0	13.9 ± 7.4	15.2 ± 6.9	13.2 ± 6.1	14.2 ± 6.4	0.877
RTI (min), mean ± SD	8.1 ± 3.7	8.0 ± 3.4	8.4 ± 2.7	8.7 ± 3.4	8.0 ± 5.3	0.354
STI (min), mean ± SD	28.1 ± 10.7	29.1 ± 9.4	30.4 ± 17.4	29.1 ± 10.0	22.5 ± 10.2	0.057
TTT (min), mean ± SD	9.5 ± 7.1	9.1 ± 6.4	10.3 ± 7.2	11.1 ± 8.0	8.8 ± 8.7	0.231
TPT (min), mean ± SD	45.5 ± 13.6	46.2 ± 11.4	49.1 ± 19.3	48.9 ± 13.7	37.8 ± 15.4	0.007
TOBC (min), mean ± SD	1.8 ± 4.5	2.5 ± 4.6	3.9 ± 6.7	-1.0 ± 3.9	1.0 ± 2.0	0.054
TOC (min), mean ± SD	10.1 ± 3.9	10.1 ± 3.6	10.3 ± 4.2	10.5 ± 4.1	9.4 ± 4.8	0.352
Outcome						
Survival to hospital admission, n (%)	81 (49.7%)	38 (38.8%)	9 (60.0%)	10 (43.5%)	24 (88.9%)	<0.001
Survival to hospital discharge, n (%)	36 (22.1%)	7 (7.1%)	4 (26.7%)	5 (21.7%)	20 (74.1%)	<0.001
Good CPC (CPC 1 or 2) at discharge, n (%)	27 (16.6%)	2 (2.0%)	2 (13.3%)	5 (21.7%)	18 (66.7%)	<0.001

Survival outcomes in prehospital RA patients after prehospital any ROSC

The survival analysis of the S→S pattern showed the highest OR (95% CI) for survival to admission, survival to discharge, and good CPC (CPC 1 or 2) at discharge irrespective of the model type (p: <0.05) (Table 3). In terms of good CPC (CPC 1 or 2) at discharge, after adjusting for age, sex, DM, occurrence site, witnessed cardiac arrest, bystander CPR, Utstein style, IV success, STI, and TPT, the ORs (95% CI) for good CPC (CPC 1 or 2) at discharge with logistic regression analysis with adjusting independent variables in the S→NS and S→S patterns were [13.61 (1.86-99.56), p=0.010 with adjusting] and [149.69 (19.51-1148.48), p: <0.001 with adjusting], respectively (Table 3). The ORs (95% CI) for good CPC (CPC 1 or 2) at discharge for STI and occurrence site on the road when a public building was set as the reference were [0.91 (0.84-0.98), p=0.015 with adjusting] and [0.02 (0.00-0.77), p=0.037 with adjusting], respectively (p: <0.05) (Table 3).

**Table 3:** Logistic regression analysis for survival outcomes in prehospital RA patients after prehospital any ROSC *Logistic regression analysis with no correction: crude model; **logistic regression analysis with adjusting for independent variables; ^†^survival to hospital admission adjusted for age, witnessed cardiac arrest, Utstein style, IV success, STI, and TPT; ^‡^survival to hospital discharge adjusted for age, sex, DM, witnessed cardiac arrest, Utstein style, IV success, STI, and TPT; ^¶^good CPC at discharge adjusted for age, sex, DM, occurrence site, witnessed cardiac arrest, bystander CPR, Utstein style, IV success, STI, and TPT OR: odds ratio; CI: confidence interval; NS: non-shockable; S: shockable; TPT: total prehospital time (min); STI: on-scene time interval (min); DM: diabetes mellitus; IV: intravenous; RA: re-arrest; ROSC: return of spontaneous circulation; CPC: cerebral performance category; CPR: cardiopulmonary resuscitation

Dependent variable	Logistic regression analysis with no correction*				Logistic regression analysis with adjusting**		
		OR (95% CI)	P-value			OR (95% CI)	P-value
Survival to hospital admission^†^	Rhythm				Rhythm		
NS→NS				1 (reference)						NS→NS	1 (reference)	
		NS→S	2.37 (0.78–7.19)		0.128		NS→S	2.82 (0.84–9.56)	0.095		
	S→NS	1.22 (0.48–3.05)	0.678		S→NS	0.92 (0.32–2.63)	0.875
	S→S	12.63 (3.56–44.85)	<0.001		S→S	7.29 (1.96–27.10)	0.003
					STI	0.94 (0.90–0.97)	0.001
					Age	0.98 (0.95–1.00)	0.068
Survival to hospital discharge^‡^	Rhythm				Rhythm		
	NS→NS	1 (reference)			NS→NS	1 (reference)	
	NS→S	4.72 (1.19–18.75)	0.027		NS→S	2.31 (0.47–11.33)	0.302
	S→NS	3.61 (1.03–12.66)	0.045		S→NS	1.64 (0.35–7.70)	0.531
	S→S	37.14 (11.71–117.78)	<0.001		S→S	13.85 (3.69–51.97)	<0.001
					Utstein style	3.95 (1.22–12.80)	0.022
					STI	0.93 (0.88–0.98)	0.009
Good CPC (CPC 1 or 2) at discharge^¶^	Rhythm				Rhythm		
	NS→NS	1 (reference)			NS→NS	1 (reference)	
	NS→S	7.39 (0.96–57.01)	0.055		NS→S	8.83 (0.86–90.60)	0.067
	S→NS	13.33 (2.40–74.12)	0.003		S→NS	13.61 (1.86–99.56)	0.01
	S→S	96 (19.14–481.60)	<0.001		S→S	149.69 (19.51–1148.48)	<0.001
					STI	0.91 (0.84–0.98)	0.015
					Occurrence site		
					Public building	1 (reference)	
					Other places	0.16 (0.01–4.79)	0.291
					Road	0.02 (0.00–0.77)	0.037
					Industrial facility	0.07 (0.00–9.11)	0.278
					Commercial facility	1.21 (0.03–64.57)	0.923
					Leisure facility	0.19 (0.00–218.01)	0.64
					Nursing facility	1.30 (0.03–64.57)	0.895
					Home (parking lot included)	0.06 (0.00–1.48)	0.086

## Discussion

The cardiac arrested patients who showed initial shockable and following shockable rhythm after ROSC were revealed to have the highest correlation with all good survival outcomes compared to those in initial non-shockable and following non-shockable rhythm after ROSC in the prehospital environment of this study. However, at the same time, the good neurologic outcome showed an inverse relationship as the number of patients with cardiac arrest occurring on the road increased compared to public buildings and as the length of time that the paramedics stayed in the field increased. The authors expect that EMS persons and physician instructors will be able to contribute to improving the patient's good survival outcome in the future if they share information like these with each other or make decisions with these in mind when treating OHCA patients. The most common cause of cardiogenic OHCA is VF [6]. Furthermore, 80-90% of patients who experience cardiac arrests due to cardiogenic causes are estimated to have VF at collapse [6-8]. The occurrence of "RA after prehospital ROSC" in OHCA patients per se allows us to predict poor survival outcomes.

Regarding the group with "no RA after prehospital ROSC", it showed the highest rates concerning witnessed cardiac arrest, Utstein style, and good survival outcomes compared with the "no prehospital ROSC" group and the "RA after prehospital ROSC" group. This result was partially consistent with a previous study that showed that witnessed OHCA in VF patients who received bystander CPR was associated with the best survival outcomes [6].

The ratio of asystole to pVT among the 163 patients increased from initial arrest rhythm to RA rhythm, and that of PEA and VF decreased. A previous study has shown that almost 50% of RAs resulted in an NS rhythm, leading to a lack of effective treatment of PEA and asystole [1]. Salcido et al. have reported the frequency of RA in non-traumatic OHCA patients in the order of PEA, VT, VF, and asystole. They have also indicated that early EMS rhythms could predict RA shockability [1]. The inconsistencies in RA rhythm results could be due to demographic, geographic, social, cultural, and/or economic differences among patients. This finding is supported by studies in which the incidence of RA varied from region to region and was inversely related to patient survival [1,9,10].

In the present study, the proportion of males was higher than that of females in all groups regardless of prehospital ROSC or RA. This is consistent with previous studies, where OHCA incidence was more prevalent in men than in women [6].

The current study suggests that the more EMS persons spend time to stay on the scene for resuscitation upon RA, the less opportunity there is for good survival outcomes. However, under SALS, due to the fact that achieving ROSC is regarded as the top priority in the field, there is a tendency to have longer on-site stays for EMS persons compared with EMS persons belonging to the conventional EMS system in South Korea. In addition, even if OHCA patients experience on-site ROSC, there is a possibility of RA recurrence. The results of this study showed that pattern change analysis between initial arrest rhythm and RA rhythm can help medical personnel to predict good survival outcomes. Additionally, reduced STI was an important factor in good survival outcomes. However, the pros and cons of the prolonged stay by EMS remain controversial. There are studies suggesting that advanced life support for critical care, such as epinephrine use or tracheal intubation, in patients with OHCA is unlikely to increase good survival outcomes such as with overall rates of ROSC or survival to hospital admission or survival to hospital discharge according to systematic review [11-13]. On the other hand, some studies have suggested that the solution to RA management in the prehospital stage is not to focus on transferring patients quickly but to monitoring the patient's condition and maintaining appropriate treatment at the time of RA, including a paradigm shift from the therapeutic point of view as such from "the scoop and run attitude" to "respond to critical situation actively" (e.g., if there is a sign of impending RA, then immediate CPR with resuscitative drug use should be resumed) [14-16].

Limitations and advantages

There are several limitations to this study. Firstly, sample data from specific geographical areas were used along with the EMS protocol, and the organization and proficiency reflect the local area and therefore cannot be generalized. Second, as a retrospective study, this study may have a frequent absence of data on potential confounding factors. However, the advantage of this study was that it was designed to be applicable to the Utstein Resuscitation Registry Templates. In addition, CPR recording paper (or SALS chart), which is configured in intervals of one minute, can be used for recording up to 60 minutes of treatment. If it exceeds 60 minutes, the paramedic and physician instructor record it online in the text field in this SALS chart, and report it orally to the senior institution manager who manages the record. Thus, records on cardiopulmonary status, resuscitation progress, ROSC, defibrillation, drug infusion, and other events are displayed for every minute according to the time of day so that the situation can be evaluated as accurately as possible. Therefore, EMS personnel may decide on how long they do the scene CPR in RA situations by SALS on remote OHCA patients in the background of this study. Finally, this study may provide an impetus for the next RA study for optimizing scene CPR time duration setup on remote OHCA patients.

## Conclusions

The cardiac arrested patients who showed initial shockable and following shockable rhythm after ROSC were revealed to have the highest correlation with all good survival outcomes such as with survival to hospital admission, survival to hospital discharge, and good CPC (CPC 1 or 2) at discharge compared to those in initial non-shockable and following non-shockable rhythm after ROSC in the prehospital environment of this study. However, at the same time, the good survival outcome showed an inverse relationship as the number of patients with cardiac arrest occurring on the road increased compared to public buildings and as the length of time that the paramedics stayed in the field increased. Verifying changes in initial cardiac arrest rhythm and prehospital RA rhythm patterns after prehospital ROSC can help us to predict good survival outcomes in the OHCA setting.
